# Isolation and partial characterisation of a novel *Trypanosoma* from the tick *Ixodes ricinus*

**DOI:** 10.1016/j.ttbdis.2020.101501

**Published:** 2020-09

**Authors:** Lisa Luu, Kevin J. Bown, Ana M. Palomar, Mária Kazimírová, Lesley Bell-Sakyi

**Affiliations:** a Institute of Infection, Veterinary and Ecological Sciences, University of Liverpool, 146 Brownlow Hill, Liverpool L3 5RF, UK; b School of Science, Engineering and Environment, G32 Peel Building, University of Salford, Salford M5 4WT, UK; c Centre of Rickettsiosis and Arthropod-borne Diseases, CIBIR, C/ Piqueras, 98, Logroño 26006, La Rioja, Spain; d Institute of Zoology, Slovak Academy of Sciences, Dubravska cesta 9, SK-84506 Bratislava, Slovakia; e The Pirbright Institute, Ash Road, Pirbright, Woking, Surrey GU24 0NF, UK

**Keywords:** *Ixodes ricinus*, Tick cell line, Trypanosome, Slovakia

## Abstract

Trypanosomes have long been recognised as being amongst the most important protozoan parasites of vertebrates, from both medical and veterinary perspectives. Whilst numerous insect species have been identified as vectors, the role of ticks is less well understood. Here we report the isolation and partial molecular characterisation of a novel trypanosome from questing *Ixodes ricinus* ticks collected in Slovakia. The trypanosome was isolated in tick cell culture and then partially characterised by microscopy and amplification of fragments of the 18S rRNA and 24Sα rDNA genes. Analysis of the resultant sequences suggests that the trypanosome designated as *Trypanosoma* sp. Bratislava1 may be a new species closely related to several species or strains of trypanosomes isolated from, or detected in, ticks in South America and Asia, and to *Trypanosoma caninum* isolated from dogs in Brazil. This study highlights the potential involvement of ixodid ticks in the epidemiology of trypanosomes, as well as the use of tick cell lines for isolation of such tick-borne protozoa. Further studies are required to investigate the epidemiology, transmission and life cycle of this putative novel species.

## 1 Introduction

Trypanosomes are protozoan parasites recognised as the causative agents of numerous important human and livestock diseases, such as Chagas' disease caused by *Trypanosoma cruzi*, African sleeping sickness caused by *Trypanosoma brucei* and surra caused by *Trypanosoma evansi* (Haag et al., 1998). Trypanosomes have been associated with infection in all vertebrate groups, including amphibians and fish (Grybchuk-Ieremenko et al., 2014; Ferreira et al., 2015; Spodareva et al., 2018), and typically demonstrate a degree of specificity with regards to host and vector (Hamilton et al., 2007; Cooper et al., 2017). Whilst transmission is frequently *via* insect vectors, other blood-feeding organisms have also been shown to be vectors, including leeches (Haag et al., 1998).

Ticks transmit a wider range of infectious agents than any other arthropod vector (Jongejan and Uilenberg, 2004) but their role as vectors of trypanosomes is less well-documented. Ticks of the genus *Ixodes* are notorious as vectors of a broad range of viral, bacterial and protozoan pathogens to livestock, companion animals and humans in many parts of the world (Jongejan and Uilenberg, 2004; Sonenshine, 2005). As well as historic studies reporting the detection or isolation of trypanosomes from *Ixodes* spp. ticks (Cunningham, 1974; Rehacek et al., 1974; Aeschlimann et al., 1979; Mungomba et al., 1987), a more recent study suggested the possibility of transmission of trypanosomes harboured by the Australian species *Ixodes australiensis via* tick faeces (Austen et al., 2011).

Here we report the short-term cultivation in a tick cell line, and partial morphological and molecular characterisation, of a novel trypanosome isolated from questing *Ixodes ricinus* ticks collected in Slovakia.

## 2 Materials and methods

### 2.1 Ticks and inoculation of tick cell cultures

Host-seeking adult male and female *I. ricinus* ticks were collected from the vegetation in the campus of the Slovak Academy of Sciences (SAS), Bratislava, Slovakia (48.17 °N, 17.07 °E; altitude *circa* 190 m above sea level). The SAS campus is a fenced area of 32 ha located on the south-western foothills of the Small Carpathian Mountains. Patches of the original oak-hornbeam forest with admixture of beech, ash, black locust, maple, lime, elm, alder, common hazel and elder are fragmented by roads, pavements and built-up areas (Kazimírová et al., 2016).

Nineteen male and 26 female ticks were collected in June 2013. Their species identity was confirmed by microscopic examination and the ticks were transferred to The Pirbright Institute where they were incubated at 15 °C, 100 % relative humidity and processed as described previously (Bell-Sakyi et al., 2015) within 5 days of collection. Briefly, the ticks were surface-sterilised in 0.1 % benzalkonium chloride for 5 min and 70 % ethanol for 1 min, then rinsed in sterile deionised water. The ticks were allowed to dry on sterile filter paper and then three pools of 6–7 male ticks and six pools of 4–5 female ticks were macerated in 1 mL Hanks' balanced salt solution. The tissue suspension was collected by pipetting and 0.2–0.3 mL aliquots were inoculated into cultures of the tick cell lines IRE/CTVM19, IRE/CTVM20 (Bell-Sakyi et al., 2007), IRE11 (Simser et al., 2002) and/or ISE6 (Kurtti et al., 1996) grown in medium with antibiotics at 28–32 °C as described previously (Bell-Sakyi et al., 2015). Cultures were monitored every few days by inverted microscope for presence of contamination, and at day 7 post inoculation (p.i.) by preparation of Giemsa-stained cytocentrifuge smears, examined using a Zeiss AxioSkop 2 Plus microscope at ×1000 oil immersion. When tick-borne microorganisms were detected by microscopy, supernatant medium was passaged onto fresh cultures of the same cell line and monitoring continued as above.

### 2.2 Morphometric analysis

Morphometric analysis was performed by light microscopy on trypanosomes in a Giemsa-stained cytocentrifuge smear using a Zeiss Imager.M2 AX10 microscope and Zen Blue acquisition software. Using ImageJ (v. 1.48) (NIH, USA), the following measurements were recorded; total length (TL), posterior end to kinetoplast (PK), kinetoplast to middle of nucleus (KN), middle of nucleus to anterior end (NA), free flagellum (FF), posterior end to middle of nucleus (PN), nucleus diameter (NL) and kinetoplast length (K). The mean and standard deviation of each measured parameter were calculated.

### 2.3 Cryopreservation and DNA extraction

The trypanosome-infected tick cell culture was resuspended and centrifuged at 1000 × *g* for 5 min, the supernate was discarded and the pellet was resuspended in 3 mL of complete L-15 medium (Bell-Sakyi et al., 2015) with 10 % glycerol, equilibrated for 20 min at room temperature, then divided between three cryovials and transferred to the vapour phase of a liquid nitrogen refrigerator. For DNA extraction, a vial was thawed rapidly by immersion in a 37 °C water bath and equilibrated for 20 min at room temperature, the contents were diluted in 9 mL complete L-15 medium and centrifuged at 1000 × *g* for 5 min and the pellet was processed for DNA extraction using a DNeasy Blood and Tissue Kit (Qiagen) following the manufacturer’s instructions for cultured cells.

### 2.4 Molecular characterisation and phylogenetic analyses

To characterise the trypanosome, two partially overlapping ∼900 bp fragments of the 18S rRNA gene, covering a ∼1600 bp section of the gene, were amplified from extracted DNA using two nested PCRs as described by McInnes et al. (2009, 2011). A fragment of the 24Sα rDNA gene with an expected product size of 265 bp was amplified according to published protocols (Souto et al., 1999). DNA extracted from *Trypanosoma congolense* (kindly provided by Dr Andrew Jackson, University of Liverpool) was used as a positive control for molecular analysis. Attempts were made to amplify fragments of the cytochrome *b* and glyceraldehyde 3-phosphate dehydrogenase (GAPDH) genes following published protocols (Barnabe et al., 2003; Hamilton et al., 2004). The PCR products were purified using a Monarch® PCR Clean Up kit (New England Biolabs, USA). Sequencing was performed in both directions by a commercial sequencing service (Source Bioscience, Nottingham, UK). The resultant sequences were edited using Bioedit v.2.7.6 software (Hall, 1999) and consensus sequences produced. Initial characterisation of both sequences was done *via* Nucleotide BLAST (GenBank, USA). The resultant sequences were submitted to GenBank through BankIt (https://www.ncbi.nlm.nih.gov/WebSub/). Phylogenetic analyses were conducted with MEGA version X using the maximum likelihood, based on the general time-reversible + G+I model, and maximum parsimony methods and including all sites (Tavaré, 1986; Hall, 2013; Kumar et al., 2018). The nucleotide substitution model was selected according to the Akaike information criterion (Akaike, 1974) implemented in Mega X. Confidence values for individual branches of the resulting trees were determined by bootstrap analysis with 500 replicates. Two phylogenetic trees were generated, one from all the available *Trypanosoma* spp. sequences with length covering almost all of the 18S rRNA fragment obtained in the present study, and one from the 18S rRNA sequences of *Trypanosoma* spp. closely related to the *Trypanosoma* sp. isolated in the present study, regardless of their length. Some of the latter sequences were too short (between 332 and 658 bp) to be included in the first analysis. The sequences used for the analyses were obtained from GenBank and aligned using the MUSCLE algorithm.

## 3 Results

### 3.1 Trypanosome cultivation

Extracts of pooled male or female ticks were inoculated into a total of 24 cultures from 2-4 different tick cell lines. In one of these extracts, derived from a pool of six male ticks, live trypanosomes were seen in the inoculum added to four different cell lines; three of the cultures became grossly contaminated with bacteria and/or fungi within the first few days. In the fourth, an ISE6 culture incubated at 32 °C, many trypanosomes were visible on day 7 p.i. when supernate was subcultured onto a fresh ISE6 culture. Both ISE6 cultures became contaminated with environmental bacteria from the inoculum, and on day 10 p.i. the less-heavily contaminated daughter culture was cryopreserved.

### 3.2 Trypanosome morphology

**Fig. 1:**
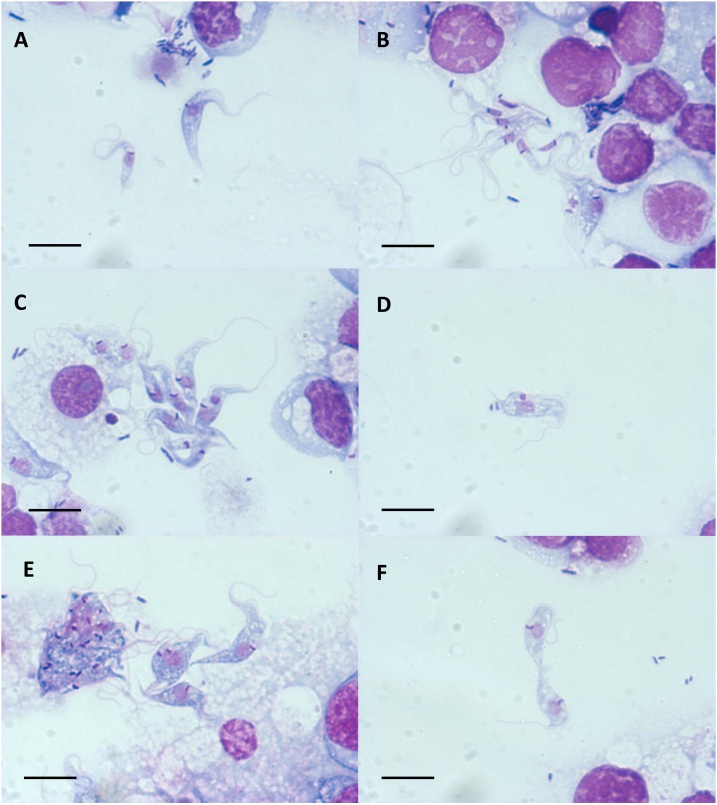
Trypanosomes isolated from a pool of tissues from six unfed adult male *Ixodes ricinus* ticks cultivated with ISE6 cells for 10 days at 32 °C. Contaminating rod-shaped bacteria can be seen. A. Epimastigote forms of varying size. B. Long slender epimastigote forms. C. Large epimastigotes and forms intermediate between epimastogote and trypomastigote (posterior kinetoplast). D. Stumpy epimastigote form. E. Large epimastigotes and aberrant form with multiple flagella, nuclei and kinetoplasts. F. Epimastigotes possibly in the final stage of division. Giemsa-stained cytocentrifuge smear viewed in a Zeiss AxioSkop 2 Plus microscope at ×1000 oil immersion and photographed with a CCD digital camera and Zeiss Axiovision software; scale bars =10 μm.

Morphology and size of the cultured trypanosomes in Giemsa-stained cytocentrifuge smears prepared after 10 days *in vitro* was quite variable (Fig. 1A), with long slender forms (Fig. 1B), intermediate forms (Fig. 1C) and shorter, stumpy forms (Fig. 1D–F) as well as large, aberrant forms with multiple flagella, nuclei and kinetoplasts (Fig. 1E). Trypanosomes possibly in the final stage of division were seen (Fig. 1F). All trypanosomes were flagellated and the kinetoplast was generally situated either just anterior to, beside or just posterior to the nucleus. Following the terminology of Hoare (1966), the cultured trypanosomes could be described as epimastigotes with a few forms transitional between epimastigote and trypomastigote. Their dimensions as determined from the stained cytocentrifuge smears were quite variable: length 30−51 μm and width 2–5 μm.

Morphometric analysis was performed to compare the isolate, designated *Trypanosoma* sp. Bratislava1, with other tick-associated *Trypanosoma* species for which equivalent data for culture-derived epimastigotes was available, namely *Trypanosoma rhipicephalis* isolated from *Rhipicephalus microplus* (Marotta et al., 2018a) and *Trypanosoma amblyommi* isolated from *Amblyomma brasiliense* (Marotta et al., 2018b) (Table 1). For all three trypanosomes, the mean values for all measured parameters except the distance from the middle of the nucleus to the anterior end (NA) and the length of free flagellum (FF) fell within the same range. For NA, *Trypanosoma* sp. Bratislava1 was shortest and *T. amblyommi* was longest, while for FF *T. rhipicephalis* was shortest and *Trypanosoma* sp. Bratislava1 was longest (Table 1).

**Table 1:** Morphometric data obtained for *Trypanosoma* sp. Bratislava1 epimastigotes compared to other tick-associated *Trypanosoma* species grown in culture.

	TL	PK	KN	NA	FF	PN	NL	K
*Trypanosoma* sp. Bratislava1	40.66 ± 4.96 (30.57–50.71)	10.71 ± 2.96 (5.59–20.34)	2.14 ± 1.61 (1.00–9.18)	8.30 ± 2.30 (2.17–12.34)	21.34 ± 3.02 (14.26–27.98)	10.55 ± 2.10 (7.27–16.00)	2.58 ± 0.63 (1.52–4.36)	1.11 ± 0.45 (0.63–2.96)
*Trypanosoma rhipicephalis*	32.44 ± 4.13	13.29 ± 2.57	1.74 ± 1.60	13.34 ± 2.97	6.90 ± 2.67	11.90 ± 2.17	1.90 ± 0.43	1.21 ± 0.36
*Trypanosoma amblyommi*	41.72 ± 8.85	15.47 ± 4.26	1.18 ± 0.38	15.61 ± 5.12	10.74 ± 2.90	14.59 ± 4.09	1.84 ± 0.37	1.23 ± 0.39

Morphometric data (μm) for *Trypanosoma* sp. Bratislava1 epimastigotes (n = 23) were compared to published data from other T*rypanosoma* species isolated from ticks, *Trypanosoma rhipicephalis* (Marotta et al., 2018a) and *Trypanosoma amblyommi* (Marotta et al., 2018b). Parameters measured were total length (TL); posterior end to kinetoplast (PK); kinetoplast to middle of nucleus (KN); middle of nucleus to anterior end (NA); free flagellum (FF); posterior end to middle of nucleus (PN); nucleus diameter (NL); kinetoplast length (K). Data are presented as mean ± standard deviation, with the range in brackets.

### 3.3 Phylogenetic analysis

**Fig. 2:**
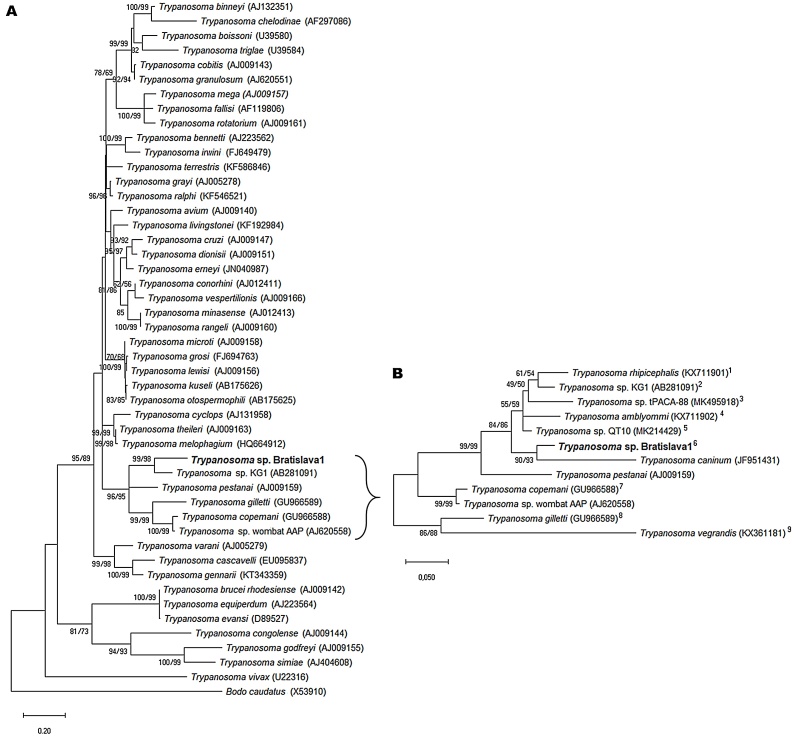
Phylogenetic analysis of *Trypanosoma* sp. Bratislava1 (in bold) and published sequences from other trypanosome species. Maximum likelihood trees were based on alignment of published 18S rRNA sequences from valid trypanosome species, and trypanosome strains that have been found in ticks. Phylogenies were inferred using the maximum likelihood and general time reversible + G+I (ML) and maximum parsimony (MP) methods (500 iterations). Numbers at the nodes are MP/ML inference support values (those <50 % are not shown). The trees are drawn to scale, with branch lengths measured in the number of substitutions per site. The GenBank accession numbers of the sequences used in these analyses are shown in brackets following each trypanosome species or strain. (A) Phylogenetic tree based on 18S rRNA sequence analysis of 48 nucleotide sequences with a total of 2020 positions in the final dataset; *Bodo caudatus* is used as an outgroup. (B) Phylogenetic tree based on 18S rRNA sequence analysis of 12 nucleotide sequences from trypanosomes closely related to *Trypanosoma* sp. Bratislava1 with a total of 1724 positions in the final dataset; tick species (geographical origin) of tick−derived trypanosomes are indicated by superscript numbers as follows: ^1^*Rhipicephalus microplus* (Brazil), ^2^*Haemaphysalis hystricis* (Japan), ^3^*Hyalomma anatolicum* (Pakistan), ^4^*Amblyomma brasiliense* (Brazil), ^5^unspecified tick (China), ^6^*Ixodes ricinus* (Slovakia), ^7^*Ixodes australiensis*, *Ixodes holocyclus* and *Ixodes tasmani* (Australia), ^8^*I. holocyclus* and *I. tasmani* (Australia), ^9^*I. holocyclus* and *I. tasmani* (Australia).

Initial phylogenetic analysis based on the 1659 bp 18S rRNA gene fragment, created by aligning the two trimmed nested PCR products, indicated that *Trypanosoma* sp. Bratislava1 was most similar to *Trypanosoma* sp. KG1 isolated from *Haemaphysalis hystricis* ticks in Japan (Thekisoe et al., 2007) (Fig. 2A). Further analysis of closely-related 18S rRNA sequences, including sequences from trypanosomes isolated from, or detected in, ticks regardless of length (Fig. 2B), confirmed that *Trypanosoma* sp. Bratislava1 was most closely-related to *Trypanosoma caninum* isolated from dogs in Brazil (Madeira et al., 2014). These two trypanosomes formed a cluster with other parasites from ticks: *T. amblyommi*, *T. rhipicephalis, Trypanosoma* sp. KG1, *Trypanosoma* sp. tPACA-88 detected in a *Hyalomma anatolicum* tick removed from a bovine in Pakistan (Zeb et al., 2019) and *Trypanosoma* sp. QT10 detected in a tick of unspecified species and life cycle stage in China (GenBank accession no. MK214429).

Characterisation based on nucleotide BLAST of a 215 bp trimmed section of the 24Sα rDNA gene PCR product with available sequences from other trypanosome species indicated that *Trypanosoma* sp. Bratislava1 was most similar to *T. rhipicephalis* (accession number KY292287; 98.7 % identity; 69 % query cover) and *T. caninum* (accession number FJ801040.1; 99.3 % identity; 60 % query cover). At the time of writing, there were no 24Sα rDNA sequences published for the other tick-related trypanosomes included in the 18S rRNA analysis. Attempts to amplify fragments of the cytochrome *b* and GAPDH genes from *Trypanosoma* sp. Bratislava1 were unsuccessful.

The 18S rRNA and 24Sα rDNA nucleotide sequences obtained from *Trypanosoma.* sp. Bratislava1 were deposited in GenBank under accession numbers MT482752 and MK558854 respectively.

## 4 Discussion

The importance of ticks in the transmission of trypanosomes has been documented relatively infrequently in comparison to insect vectors. We report here the first partial characterisation of a *Trypanosoma* sp. isolated from a European tick. Whilst trypanosomes have previously been cultured from *I. ricinus* collected in Scotland (Cunningham, 1974), and detected in the haemolymph of ticks from Austria, Slovakia, Switzerland and Wales (Rehacek et al., 1974; Aeschlimann et al., 1979; Mungomba et al., 1987), molecular tools were not available to characterise those parasites. In the Swiss study, five of 2501 ticks showed evidence of infection in their haemolymph (Aeschlimann et al., 1979); in the Welsh study one of 325 questing nymphs crushed individually and examined by wet preparation was trypanosome-positive (Mungomba et al., 1987), whilst in the Scottish study trypanosomes were cultured in one of nine individual partially-engorged adult tick explants (Cunningham, 1974). The present study reports a single isolate from a pool of six male ticks within a sample of 45 questing ticks of both sexes and, combined with the paucity of studies reporting trypanosomes in *I. ricinus,* our results strongly suggest that infection of this tick species is relatively rare.

The majority of the developmental forms of *Trypanosoma* sp. Bratislava1 cultured with tick cells at 32 °C resembled epimastigotes (kinetoplast beside or just anterior to the nucleus), with a few forms intermediate between epimastigote and trypomastigote (kinetoplast posterior to the nucleus) (Hoare, 1966). This is in agreement with the observation of Cunningham (1974) who reported “a shimmering mass of epimastigotes” in a 6-week-old explant culture comprising the entire body contents of a partially-fed adult *I. ricinus* tick removed from a cow on the Isle of Raasay, Scotland. Similarly, Aeschlimann et al. (1979) described epimastigote and trypomastigote trypanosomes in the haemolymph of Swiss adult *I. ricinus* ticks, and Mungomba et al. (1987) reported epimastigotes in unfed nymphal *I. ricinus* from a sheep-grazing area in North Wales. The size range of *Trypanosoma* sp. Bratislava1 (length 30−51 μm, width 2–5 μm) overlapped with that reported for trypanosomes detected in Welsh ticks (length 24–33 μm, width 1–4 μm) (Mungomba et al., 1987); the latter measurements were obtained from Giemsa-stained nymphal crush smears, in which the parasites may have been less fully spread out than our trypanosomes in cytocentrifuge smears, resulting in apparently smaller dimensions.

Morphometric comparison of *Trypanosoma* sp. Bratislava1 with trypanosomes isolated from other tick species in Brazil revealed that while most measured parameters were similar, all three species differed considerably in the distance from the nucleus to the anterior end and the length of free flagellum, indicating that *Trypanosoma* sp. Bratislava1 was unlikely to belong to either of the two Brazilian trypanosome species.

The influence of a period of *in vitro* culture (incubation temperature, origin of feeder cells) on the range of developmental forms exhibited by tick-derived trypanosomes is evident from our study and those of previous authors. Thekisoe et al. (2007) described trypomastigote (typical vertebrate bloodstream) forms in *Trypanosoma* sp. KG1 propagated with human HEK 293 cells at 37 °C, whereas when these parasites were inoculated into *Ornithodoros moubata* ticks incubated at 25 °C, both trypomastigote and epimastigote forms were seen. Marotta et al. (2018a, 2018b) reported a predominance of epimastigote (typical arthropod) forms in both *T. rhipicephalis* and *T. amblyommi* over the first 10 days of propagation with *I. scapularis* tick cells at 30 °C followed by an increase in spheromastigote forms. In our study, we similarly observed a predominance of epimastigote forms after 10 days cultivation of *Trypanosoma* sp. Bratislava1 with *I. scapularis* cells at 32 °C; unlike the previous authors (Marotta et al., 2018a, 2018b) we did not observe typical spheromastigote forms, though we did see aberrant forms with multiple nuclei, kinetoplasts and flagella, possibly resulting from a failure to complete cell division *in vitro*.

Phylogenetic analyses suggest that *Trypanosoma* sp. Bratislava1 isolated from *I. ricinus* is closely related to *T. caninum*. This species has been identified in Brazilian dogs, where the infection is largely asymptomatic and results in a minor humoral immune response (Madeira et al., 2014). The vector of this species is unknown, although it appears not to be triatomine insects (Madeira et al., 2014). Of the other similar trypanosomes, *Trypanosoma* sp. KG1, *T. rhipicephalis* and *T. amblyommi* were all isolated from ticks (Thekisoe et al., 2007; Marotta et al., 2018a, 2018b) while *Trypanosoma* sp. tPACA-88 (Zeb et al., 2019) and *Trypanosoma* sp. QT10 were detected in ticks. This suggests that it would be interesting to explore the potential for ticks to act as vectors of *T. caninum* and whether this trypanosome clade is associated with transmission by ixodid ticks. Other, less closely-related species of trypanosome have also been associated with tick transmission. Latif et al. (2004) fed nymphal *H. anatolicum* ticks on a calf with a *Trypanosoma theileri* parasitaemia; following moult, nearly half of the resultant adult ticks showed high levels of trypanosomes in haemolymph smears, although onward transmission was not attempted. When unfed adult trypanosome-infected *H. anatolicum* collected in the field in Sudan were allowed to feed on experimental calves, one of two animals showed trypanosomes in a Giemsa-stained biopsy smear prepared from the local drainage lymph node on day 5 post application, and trypanosomes were re-isolated *in vitro* from this calf (Morzaria et al., 1986). In Australia *Trypanosoma copemani* has recently been associated with *I. australiensis*, as viable forms could be isolated from ticks several months after removal from their mammalian host (Austen et al., 2011). Furthermore, the authors suggested that transmission was potentially more likely to be through the oral-faecal route as viable forms were found in faecal material but not in salivary glands. However, a more recent review considered that faecal (stercorian) transmission of trypanosomes harboured by ticks was unlikely, and emphasised the need for further experimental work to determine the significance of ticks in trypanosome life cycles (Krige et al., 2019).

The life cycle of *Trypanosoma* sp. Bratislava1 is unclear, in particular regarding its natural vertebrate host. The fact that *I. ricinus* will feed on such a wide range of vertebrates, from small mammals, birds and reptiles to large mammals such as deer (Rizzoli et al., 2014), makes it difficult to predict the likely host. Similarly, the 18 s rRNA and 24Sα rDNA sequences obtained do not match any trypanosome genotypes previously obtained from European mammals or birds. Certainly, the phylogenetic analysis suggests that it is not *T. theileri,* which was suggested as the species detected in the Scottish and Swiss *I. ricinus* (Cunningham, 1974; Aeschlimann et al., 1979) or *Trypanosoma melophagium*, proposed as a possible identity for the species detected in Welsh *I. ricinus* (Mungomba et al., 1987) and more recently subjected to phylogenetic analysis (Martinkovic et al., 2012). Interestingly, attempts to broaden the phylogenetic analysis by amplifying fragments of the cytochrome *b*and GAPDH genes from *Trypanosoma* sp. Bratislava1 DNA following published protocols (Barnabe et al., 2003; Hamilton et al., 2004) were unsuccessful, suggesting that the sequences of these genes may differ substantially from those of recognised trypanosome species.

Isolation of *Trypanosoma* sp. Bratislava1 was a fortuitous by-product of a study aiming to isolate tick-borne bacteria *in vitro* (Bell-Sakyi et al., 2015); as we did not anticipate finding trypanosomes, we did not carry out any pre-cultivation screening of the ticks such as preparation of haemolymph smears. Moreover, the high incidence of contamination with environmental bacteria reported previously (Bell-Sakyi et al., 2015) unfortunately affected our trypanosome culture and prevented us from carrying out additional *in vitro* analyses. Further work is therefore required to determine if *Trypanosoma* sp. Bratislava1 represents a new species, establish its distribution and prevalence in ticks and identify its vertebrate host. Our study confirms the usefulness of tick cell lines as a substrate for isolation of trypanosomes (Marotta et al., 2018a, 2018b), as well as other tick-borne microorganisms (Kurtti et al., 1996; Simser et al., 2002; Alberdi et al., 2012; Bell-Sakyi et al., 2015; Palomar et al., 2019), from ticks collected in the field.

## CRediT authorship contribution statement

**Lisa Luu:** Formal analysis, Investigation, Methodology, Visualization, Writing - review & editing. **Kevin J. Bown:** Conceptualization, Formal analysis, Investigation, Writing - original draft, Writing - review & editing. **Ana M. Palomar:** Formal analysis, Investigation, Methodology, Visualization, Writing - review & editing. **Mária Kazimírová:** Conceptualization, Resources, Writing - original draft, Writing - review & editing. **Lesley Bell-Sakyi:** Conceptualization, Funding acquisition, Investigation, Methodology, Resources, Supervision, Writing - original draft, Writing - review & editing.
